# Reconstruction of the internal iliac artery in patients with aneurysmal disease: Two case reports

**DOI:** 10.3892/etm.2013.1459

**Published:** 2013-12-24

**Authors:** JIN HYUN JOH, HO-CHUL PARK

**Affiliations:** Department of Surgery, Kyung Hee University Hospital at Gangdong, Kyung Hee University School of Medicine, Seoul 134-727, Republic of Korea

**Keywords:** iliac artery, internal, artery bypass, endovascular procedures

## Abstract

During the endovascular repair of aortoiliac aneurysm, bilateral internal iliac artery (IIA) occlusion may give rise to significant morbidities such as ischemia of buttock or sigmoid colon and erectile dysfunction. Open and endovascular approaches are used to maintain IIA circulation in such cases. The present report describes the cases of two patients who underwent external-to-internal iliac artery bypass surgery, one via a novel hybrid approach. The first patient had a right common iliac artery aneurysm without a sufficient distal landing zone for endovascular repair. The distal landing of the device was therefore placed in the right external iliac artery (EIA). The tortuous portion of the right EIA was excised and anastomosed in an end-to-end fashion. An external-to-internal iliac artery bypass was then performed. The second patient underwent endovascular repair of the IIA aneurysm with a Gore^®^ Viabahn^®^-covered stent (W. L. Gore and Associates, Inc., Flagstaff, AZ, USA). This covered stent was anastomosed with the iliac bypass graft. Postoperative pelvic circulation was maintained. In conclusion, this strategy for maintaining IIA flow is a potential novel approach for future use.

## Introduction

Successful endovascular aneurysm repair (EVAR) requires sufficient proximal and distal landing zones that are relatively free from disease (1). If the aortoiliac aneurysm involves the iliac bifurcation, successful EVAR may be limited. In these cases, the distal landing zone should be in external iliac artery (EIA) which may interrupt the flow of internal iliac artery (IIA). Bilateral IIA occlusion during the endovascular repair of aneurysms is associated with significant morbidity, including buttock claudication, erectile dysfunction and ischemia of the sigmoid colon and perineum (2,3).

Open or endovascular approaches are used to maintain IIA circulation in such cases during EVAR. Endovascular options include the double-barrel technique (4) or the use of iliac branch devices (IBDs) (5). External-to-internal iliac artery bypass is a reasonable option for an open approach to preserve pelvic circulation (6). The present report describes a patient with a common iliac artery (CIA) aneurysm who underwent external-to-internal iliac bypass surgery, and a second patient who was treated via a novel hybrid approach to repair an IIA aneurysm.

## Case reports

### Case 1

**Figure 1:**
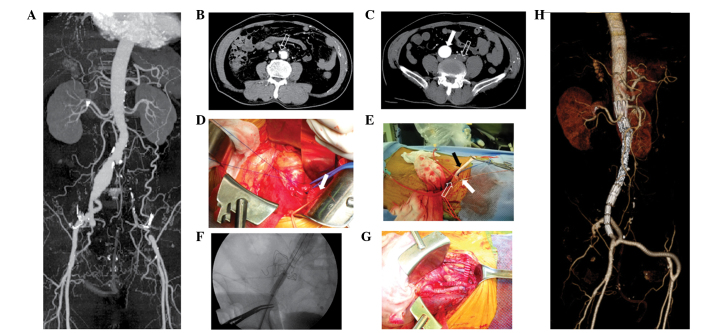
Case 1. A 70-year-old male patient had (A) a right common iliac artery (CIA) aneurysm, long-segment occlusion of the left iliac artery and (B) a penetrating atherosclerotic ulcer in the infrarenal abdominal aorta (indicated by the open arrow). (C) The axial image shows the CIA aneurysm (white arrow) and the occluded left iliac artery (white arrow). (D) The external iliac artery (EIA) was tortuous at the time of exposure. (E) Two introducer sheaths were inserted into the right EIA for angiograms (closed arrow) and for the delivery of the stent graft (empty arrow) with separate punctures. The anastomosed graft was seen in this picture (black arrow). (F) Endovascular repair of the right CIA aneurysm was successful. (G) External-to-internal iliac artery bypass, end-to-end anastomosis following the division of the tortuous EIA and extra-anatomic crossover femoral bypass were performed. (H) Postoperative computed tomography-angiography showed that the repair of the aneurysm and the patent bypassed graft was successful.

A 70-year-old male patient presented with severe claudication of the left leg. Hypertension and a cerebrovascular attack featured in the past medical history of the patient. The patient’s ankle brachial index (ABI) was 1.11 for the right leg and 0.55 for the left. Computed tomography-angiography (CTA) showed long-segment occlusion of the left iliac artery and a right CIA aneurysm with a maximal diameter of 3.2 cm (Fig. 1A and C). There were multiple penetrating atherosclerotic ulcers (PAUs) in the infrarenal abdominal aorta (Fig. 1B). Simultaneous repairs of the right CIA aneurysm and bypass surgery for the left iliac occlusion were planned. A longitudinal skin incision, ~5 cm long, was made in the left inguinal crease, exposing the left femoral artery. A second oblique skin incision was made on the right lower abdomen to approach the right iliac arteries. The right EIA was tortuous. Following the division of the right IIA, the proximal end was sealed using 4-0 Prolene™ (Ethicon, Blue Ash, OH, USA) sutures (Fig. 1D). The distal portion of the IIA was anastomosed with a 7-mm expanded polytetrafluoroethylene (ePTFE) graft. Two 7F introducer sheaths were subsequently inserted into the right EIA with separate punctures (Fig. 1E). One sheath was used for the angiogram, while the other was used for the delivery of the stent graft. A 5F pigtail catheter was inserted though the distal introducer sheath to obtain the angiogram. A 28-mm Zenith main body device and a Zenith converter (Cook, Inc., Bloomington, IN, USA) were deployed serially in the aorta and the iliac aneurysm (Fig. 1F). The distal landing of the device was placed in the right EIA. Subsequently, the tortuous portion of the right EIA was excised, prior to being anastomosed in an end-to-end fashion. The ePTFE graft, which was anastomosed with the right IIA, was connected onto the right EIA. A second 7-mm ePTFE graft was placed though the lower abdominal wall. An extra-anatomic bypass was made from the IIA bypass graft to the left femoral artery (Fig. 1G). Postoperative CTA showed that the repair of the aneurysm was successful and the bypass graft was patent (Fig. 1H).

### Case 2

**Figure 2:**
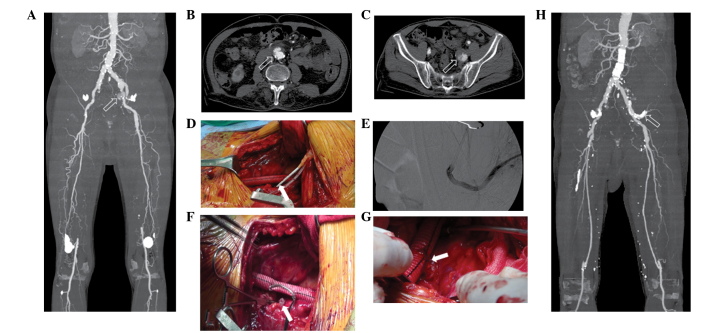
Case 2. A 69-year-old male patient had (A and C) a left internal iliac artery (IIA) aneurysm (open arrows), (A) bilateral superficial femoral artery occlusion and (B) focal aneurysmal change of the infrarenal abdominal aorta (open arrow). (D and E) Endovascular repair of the IIA aneurysm was performed with direct puncture of the aneurysm (white arrow). (F and G) A covered stent to repair the IIA aneurysm was anastomosed with the iliac bypass graft (white arrows). (H) Postoperative computed tomography-angiography showed that there were no endoleaks and that the covered stent graft to repair the IIA aneurysm was patent (open arrow).

A 69-year-old male patient presented with severe claudication of his bilateral lower extremities. The patient had hypertension, chronic obstructive pulmonary disease, cerebrovascular attack and atrial fibrillation, as well as an ABI of 0.56 for the right leg and 0.55 for the left. Preoperative CTA showed bilateral superficial femoral artery occlusion. A focal aneurysmal change was observed in the infrarenal abdominal aorta and a left IIA aneurysm was visualized (Fig. 2A–C). The maximal diameter of the left IIA aneurysm was 3.2 cm. A right femoropopliteal bypass was performed using the ipsilateral great saphenous vein. One month later, left femoropopliteal bypass and the repair of the infrarenal aortic aneurysm and IIA aneurysm were planned. A routine femoropopliteal bypass was prepared with the left great saphenous vein. An oblique skin incision was made on the left lower abdomen to approach the left iliac artery. Following the exposure of the left iliac artery, the left CIA was divided. Having closed the distal portion of the left CIA, a 10-mm Dacron graft (Meadox Medicals, Oakland, NJ, USA) was connected to the proximal portion of the left CIA. Two 7F introducer sheaths were then inserted into the Dacron graft. One sheath was used for a 5F pigtail catheter, which was placed for the angiograms, while the second sheath was used for the insertion of the stent graft. A 24×58 mm aortic extender of the Zenith device (Cook Inc.) was implanted into the abdominal aorta to fix the aortic aneurysm. Following the successful repair of the aneurysm, the Dacron graft was anastomosed with the distal EIA. A 7F introducer sheath was inserted into the IIA aneurysm by direct puncture (Fig. 2D). With this sheath, a 6×100 mm Gore^®^ Viabahn^®^-covered stent (W. L. Gore and Associates, Inc., Flagstaff, AZ, USA) was placed into the IIA aneurysm. The completion angiogram showed that there was no endoleak into the IIA aneurysm (Fig. 2E). A thrombin-soaked Gelfoam® (Pfizer, Brussels, Belgium) was used to fill the aneurysm. The scalloped, proximal portion of the Viabahn stent was excised. The stent was then anastomosed with the Dacron graft (Fig. 2F). Following the successful repair of the aortic aneurysm and left IIA aneurysm, routine femoropopliteal bypass was performed using the ipsilateral great saphenous vein of the patient’s left leg. Postoperative CTA showed no endoleaks at the site of aneurysm repair and a patent vein graft, as well as the Viabahn-covered stent in the IIA aneurysm.

## Discussion

The use of EVAR for the repair of aortoiliac aneurysms is steadily increasing. If a sufficient distal landing zone is not present in the CIA during EVAR, there may be a requirement to extend the iliac limbs into the EIA. Embolization of the IIA may be required to prevent type II endoleaks from the IIA. The pelvic circulation may be interrupted if embolization of the bilateral IIA is necessary. The interruption of the pelvic circulation may result in buttock ischemia, spinal cord, bowel and bladder ischemia, as well as erectile dysfunction (7). For this reason, novel endovascular approaches have recently been investigated, including the double-barrel technique (4) or the use of IBDs (5).

External-to-internal iliac artery bypass is another open approach strategy. Unno *et al* (6) described five patients who underwent external-to-internal iliac artery bypass during the endovascular repair of abdominal aortic aneurysms and bilateral CIA aneurysms (6). None of these patients had experienced new-onset erectile dysfunction or buttock claudication one month subsequent to surgery. In the present study, 7-mm ePTFE grafts were used in the patients. Since the IIA is in a deeper portion of the pelvic cavity, it is difficult to anastomose the graft and the IIA. To overcome this difficulty, the graft was connected to the IIA in an end-to-end fashion. Following the completion of the EVAR, the graft was connected to the EIA.

The EIA is smaller in diameter and more tortuous than the CIA. The deployment of endograft limbs into the EIA leads to higher rates of occlusion and leg amputation (8). Franz reported that the excision of the tortuous EIA and end-to-end anastomoses were able to overcome the difficulties associated with the tortuosity of the EIA (9). This simple procedure was performed in the first patient described in the present study. Following the deployment of the stent graft, the tortuous portion of the EIA was excised and end-to-end anastomosis was subsequently achieved using 5-0 Prolene sutures.

In the first patient described in this study, the left iliac artery was already occluded. Another route was therefore sought in order to obtain an angiogram during the placement of the stent graft. In such situations, the left brachial artery is commonly used. However, the brachial approach is associated with complications. Alvarez-Tostado *et al* (10) revealed that brachial access site-related complications occurred in 21 (6.5%) out of 323 patients (10). Thirteen of the 21 patients (62%) required surgical correction, mostly for brachial artery thrombosis or pseudoaneurysm. In the present study, it was possible to insert two introducer sheathes into the EIA in the patients, since the EIA had already been exposed. It is more comfortable for the patient for the angiogram sheath to be inserted distally, since this avoids the interruption of the pigtail catheters during the insertion of the main body.

The endovascular options for the repair of IIA aneurysms include the double-barrel technique and the use of IBDs. However, in some countries, IBDs are not available. In the second patient in the present study, the treatment plan was to repair the aortic aneurysm and bypass the stenotic segment of the left iliac artery. Therefore, a 10-mm Dacron graft was connected to the left CIA following division. This type of graft is commonly used to create iliac conduits during EVAR or thoracic endovascular aneurysm repair (11). The graft was then anastomosed to the distal EIA. A 7F introducer sheath was directly inserted into the IIA aneurysm. Using this sheath, a Viabahn-covered stent was easily inserted into the IIA aneurysm. Having confirmed that there was no endoleak, thrombin-soaked Gelfoam^®^ was used to fill the aneurysm. This covered stent was anastomosed to the bypassed Dacron graft and suturing was completed without difficulty. This approach is a feasible option for the repair of IIA aneurysms.
